# PULPO: pipeline of understanding large-scale patterns of oncogenomic signatures

**DOI:** 10.1093/bioinformatics/btag118

**Published:** 2026-03-10

**Authors:** Marta Portasany-Rodríguez, Gonzalo Soria-Alcaide, Elena G Sánchez, Mariya Ivanova, Ana Gómez, Reyes Giménez, Jaanam Lalchandani, Gonzalo García-Aguilera, Silvia Alemán-Arteaga, Cristina Saiz-Ladera, Manuel Ramírez-Orellana, Jorge Garcia-Martinez

**Affiliations:** Oncohematology Department, Fundación para la Investigación Biomédica Hospital Infantil Universitario Niño Jesús (FIBHNJS), Madrid 28009, Spain; Escuela de Doctorado, Universidad Autónoma de Madrid (UAM), Ciudad Universitaria de Cantoblanco, C/ Francisco Tomás y Valiente 2, Madrid 28049, Spain; Oncohematology Department, Fundación para la Investigación Biomédica Hospital Infantil Universitario Niño Jesús (FIBHNJS), Madrid 28009, Spain; Escuela de Doctorado, Universidad Autónoma de Madrid (UAM), Ciudad Universitaria de Cantoblanco, C/ Francisco Tomás y Valiente 2, Madrid 28049, Spain; Oncohematology Department, Fundación para la Investigación Biomédica Hospital Infantil Universitario Niño Jesús (FIBHNJS), Madrid 28009, Spain; Oncohematology Department, Fundación para la Investigación Biomédica Hospital Infantil Universitario Niño Jesús (FIBHNJS), Madrid 28009, Spain; Oncohematology Department, Hospital Infantil Universitario Niño Jesús (HNJS), Madrid 28009, Spain; Oncohematology Department, Fundación para la Investigación Biomédica Hospital Infantil Universitario Niño Jesús (FIBHNJS), Madrid 28009, Spain; Oncohematology Department, Fundación para la Investigación Biomédica Hospital Infantil Universitario Niño Jesús (FIBHNJS), Madrid 28009, Spain; Escuela de Doctorado, Universidad Autónoma de Madrid (UAM), Ciudad Universitaria de Cantoblanco, C/ Francisco Tomás y Valiente 2, Madrid 28049, Spain; Oncohematology Department, Fundación para la Investigación Biomédica Hospital Infantil Universitario Niño Jesús (FIBHNJS), Madrid 28009, Spain; Oncohematology Department, Fundación para la Investigación Biomédica Hospital Infantil Universitario Niño Jesús (FIBHNJS), Madrid 28009, Spain; Oncohematology Department, Fundación para la Investigación Biomédica Hospital Infantil Universitario Niño Jesús (FIBHNJS), Madrid 28009, Spain; Oncohematology Department, Hospital Infantil Universitario Niño Jesús (HNJS), Madrid 28009, Spain; Oncohematology Department, Instituto de Investigación Sanitaria La Princesa (IISLP), Madrid 28006, Spain; Oncohematology Department, Instituto de Investigación Sanitaria La Princesa (IISLP), Madrid 28006, Spain

## Abstract

**Summary:**

PULPO v1.0 is a novel; fully automated pipeline designed for the preprocess and extraction of mutational signatures from raw Optical Genome Mapping (OGM) data. Built using Snakemake and executed within an isolated, Conda-managed environment, PULPO transforms complex cytogenetic alterations, captured at ultra-high resolution, into Catalogue of somatic mutations in cancer mutational signatures (COSMIC). This innovative approach not only enables researchers to work directly from raw OGM inputs but also streamlines the traditionally complex process of signature extraction, making advanced oncogenomic analyses accessible to users with varying levels of bioinformatics expertise. By facilitating the integration of comprehensive structural variants (SVs) and copy number variants (CNVs) data with established signature catalogues, PULPO paves the way for improved diagnostic accuracy and personalized therapeutic strategies.

**Availability and implementation:**

The pipeline is open source and freely available under the MIT License at https://github.com/OncologyHNJ/PULPO-v.1.0 and DOI in Zenodo: https://zenodo.org/records/17749097.

## 1 Introduction

Structural variants (SVs) and copy number variants (CNVs) are major classes of genomic alterations that play pivotal roles in carcinogenesis and tumour progression (Beroukhim *et al.* 2010, Weischenfeldt *et al.* 2013, Xu *et al.* 2022). Despite their biological significance, the systematic detection of these alterations has long been restricted by the limitations of conventional cytogenetic techniques such as karyotyping, fluorescence in situ hybridization (FISH), and multiplex ligation-dependent probe amplification (MLPA) (Alías *et al.* 2011, Park *et al.* 2011). These traditional methods often lack the resolution necessary to detect small CNVs or complex rearrangements, such as chromothripsis or balanced translocations, and are frequently hampered by high costs and labour-intensive workflows.

The recent advent of OGM has transformed the landscape of genomic analysis by enabling genome-wide detection of SVs and CNVs at an ultra-high resolution (<1 kb) in a single assay (Neveling *et al.* 2021, Levy *et al.* 2024). Moreover, OGM is capable of detecting a wide range of chromosomal alterations across the entire genome, not limited to restricted areas. Studies have demonstrated that OGM offers superior sensitivity and resolution compared to standard cytogenetic methods, reporting up to a 30% increase in diagnostic yield for both hematologic malignancies and solid tumours (Neveling *et al.* 2021). This breakthrough technology not only refines the detection of SVs but also provides an unprecedented opportunity to explore the intricate molecular architecture that underpins tumorigenesis.

Beyond the detection of genomic alterations, the analysis of mutational signatures—defined as the patterns of somatic alterations left by various endogenous processes (e.g. defective DNA repair mechanisms) or exogenous exposures (e.g. tobacco smoking and UV radiation)—has emerged as a crucial tool for understanding cancer aetiology (Steele *et al.* 2022). While the mutational landscapes of single-base substitutions (SBS), doublet base substitutions (DBS), and small insertions/deletions (ID) have been extensively catalogued in resources like Catalogue of somatic mutations in cancer mutational signatures (COSMIC) (Alexandrov *et al.* 2020), structural mutational signatures associated with SVs and CNVs remain less characterized. The limited resolution of prior genomic platforms has prevented a systematic interrogation of the complex breakpoint patterns that could unveil novel insights into the mutagenic processes driving cancer (Cortés-Ciriano *et al.* 2020, Degasperi *et al.* 2020, Dentro *et al.* 2021).

The integration of OGM with mutational signature analysis represents a highly promising frontier in precision oncology. SigProfiler, widely recognized for its robust performance in identifying similarities with COSMIC signatures, has firmly established itself as a powerful tool in this domain (Bergstrom *et al.* 2019, Islam *et al.* 2022). However, its implementation necessitates specialized bioinformatics expertise and programming skills. To broaden access to mutational signature analysis and enhance usability, we have developed PULPO (Pipeline of Understanding Large-scale Patterns of Oncogenomic signatures). PULPO incorporates the similarity of search capabilities of SigProfiler within an intuitive, user-friendly framework, while also pioneering the use of OGM data. This innovation not only streamlines the analytical workflow but also offers a novel perspective for elucidating the mutagenic processes that underlie cancer development.

PULPO distinguishes itself by integrating high-resolution SV/CNV data obtained through OGM with the mutational signature repository provided by COSMIC (Alexandrov *et al.* 2020). Utilizing similarity-based algorithms, the pipeline systematically compares OGM-derived variant profiles to the COSMIC catalogue, enabling the precise identification of structural signatures. Built on a preconfigured Snakemake pipeline, PULPO streamlines data preprocessing, signature similarity scoring, and visualization, thereby democratizing access to this advanced analysis even for researchers with limited bioinformatics expertise.

The clinical implications of PULPO are substantial. By linking structural mutational signatures to specific aetiological processes and therapeutic vulnerabilities, PULPO not only enhances our understanding of the molecular drivers of cancer but also opens new avenues for translational research. For instance, matching signature profiles to therapy-associated patterns such as those related to APOBEC activity or homologous recombination deficiency can facilitate drug repositioning and the identification of actionable targets (Alexandrov *et al.* 2020, Watkins *et al.* 2023). Furthermore, recent work has demonstrated that geographic variation of mutagenic exposures can significantly impact cancer genomes (Senkin *et al.* 2024). In summary, by leveraging the transformative resolution of OGM, PULPO bridges a critical gap in structural mutational signature analysis and holds significant promise for clinical applications, ranging from improved diagnostic accuracy to the personalization of cancer treatment.

## 2 Materials and methods

**Figure 1:**
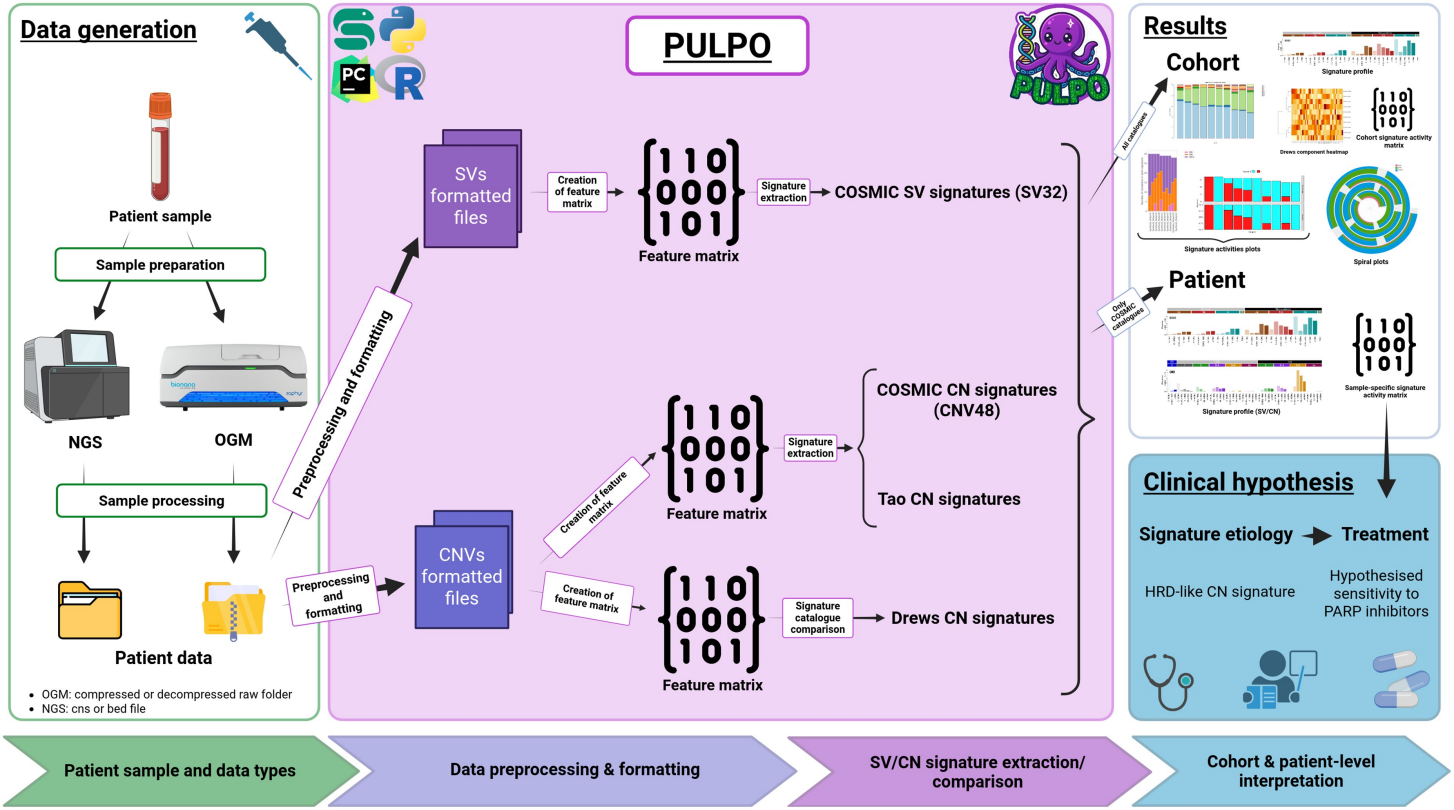
Overview of the PULPO pipeline for SV and CN mutational signature analysis. Patient tumour samples are processed and prepared for OGM or NGS, producing raw OGM folders or CNV/SV call files (e.g. CNS or BED formats). These constitute the patient data used as input for PULPO. PULPO processing and signature extraction. SV and CNV calls are pre-processed and formatted into standardized SVs/CNVs formatted files and subsequently converted into feature matrices. From SV feature matrices, PULPO extracts COSMIC SV signatures (SV32). From CN feature matrices, PULPO estimates activities of COSMIC CN signatures (CNV48) and Tao CN signatures and performs catalogue comparisons with Drews CN signatures. PULPO generates cohort-level outputs, including signature exposure profiles, cohort signature activity matrices, decomposition plots and structural landscape visualizations [e.g. spiral plots (Gu and Hübschmann 2022)], as well as patient-level SV/CN signature profiles. SV/CN signature patterns can be explored to formulate hypotheses about underlying biological processes and potential therapeutic sensitivities. As an example, an HRD-like CN signature may be associated with hypothesized sensitivity to PARP inhibitors. Created in BioRender https://BioRender.com/c6p66yz.

PULPO v1.0 was developed using Snakemake v7.32.4, a robust workflow management system that ensures scalability, reproducibility, and modularity in bioinformatics pipelines (Mölder *et al.* 2021). Its design strongly emphasizes automation and user transparency, enabling integration into high-throughput computational environments. To ensure reproducibility across platforms and users, PULPO is executed within a fully controlled and isolated Conda environment (Anaconda 2016), which includes all the necessary dependencies specified in an environment file (available on GitHub). The pipeline workflow is summarized in Fig. 1, and the main steps of the analysis are detailed below.

### 2.1. QC, filtering input files, and preprocessing

For OGM data, CNVs and SVs were first filtered within the Bionano Rare Variant pipeline which applies predefined quality, overlap and confidence thresholds (Table 1, available as supplementary data at *Bioinformatics* online). Only variants passing these filters were exported and used as raw OGM input for PULPO, ensuring that downstream signature analyses are performed on high-confidence SV and CNV calls.

PULPO accepts either a compressed raw OGM archive (.zip) or the corresponding uncompressed directory, as specified in the configpulpoOGM.yaml configuration file. SVs input is provided as smap files generated by Bionano Access, whereas CNVs input is supplied as csv or txt exports. The OGM module has been validated with Bionano Access versions 1.6.1, 1.8.1, and 1.8.3 to ensure compatibility with current and older software releases, new pipelines of Bionano could be analysed with this tool as long as the output files maintain the same data structure.

For NGS data, the workflow expects bed or cns format for CNVs and SVs calls in bedpe format. The accompanying GitHub repository provides helper scripts to convert raw outputs from commonly used callers (e.g. CNVkit, Manta and consensus seg files) into the formats required by PULPO (bed or cns), including the necessary preprocessing and harmonization steps to ensure seamless integration into the pipeline.

Before initiating any downstream analysis, PULPO performs a rigorous quality control step by validating input files to ensure that no sample is empty. If an empty sample file is detected, the pipeline halts execution immediately and generates an informative error log, automatically stored in the designated log directory within the user-specified working folder.

The data conversion module takes raw OGM data and transforms it into a format suitable for the SigProfiler tool suite. Specifically, it converts OGM-derived CNV data into bed format and SV data into bedpe format, both widely used and standardized formats in numerous bioinformatics tools. Since OGM files are converted into bed and bedpe formats, the tool can alternatively be initialized directly with these standard file types from NGS (after here is where the formatted NGS data starts in the pipeline).

### 2.2 Decomposition and signature extraction

#### 2.2.1 Steele

Once the data are formatted, they are processed using SigProfilerMatrixGenerator v1.2.28 and SigProfilerExtractor v1.1.24 (Islam *et al.* 2022, Khandekar *et al.* 2023). The pipeline offers two main modes of analysis:

1. Per-sample analysis for individual-level signature exploration.
2. Combined cohort + sample analysis to facilitate broader comparative analyses while preserving individual resolution.

By default, PULPO employs the GRCh38 human reference genome to ensure consistency; however, its modular architecture allows future extensions to other reference builds, based on user requirements. To enhance usability and flexibility, several key parameters of the SigProfilerExtractor tool, such as minimum_signatures, maximum_signatures, and nmf_replicates, can be explicitly defined by the user or selected automatically using optimized, dataset-specific default settings, thereby lowering the entry barrier for less experienced users while permitting fine-tuning for advanced applications.

PULPO enables the analysis of SVs and CNVs in COSMIC CN and SV signatures either independently or in combination, offering a versatile and customizable approach for researchers interested in exploring the full spectrum of large-scale genomic rearrangements and their associated mutational processes. This makes the tool particularly valuable in cancer genomics studies, where such alterations play a key role. We selected the COSMIC CN signature framework (Steele *et al.* 2022) computed using SigProfilerMatrixGenerator and SigProfilerExtractor (Bergstrom *et al.* 2019, Islam *et al.* 2022, Khandekar *et al.* 2023) as the main CN signature analysis method in PULPO because it is currently the gold standard in the field (Tao *et al.* 2023).

#### 2.2.2 Additional methodologies for CNVs

Drews *et al.* (2022) and Tao *et al.* (2023) methods were implemented in the study of CNVs to give a most extended overview and a comparative between catalogues. Although there are two different techniques, the implementation has similarities. A specific module of preprocessing and formatting CNVs files were constructed as a first step of the analysis similar to the preprocessing of CNVs in the Steele methodology to adapt the CNVs inputs to the specific format requirements of each method. These additional methodologies were applied at the cohort level only, as both Drews *et al.* (2022) and Tao *et al.* (2023) report more robust performance and more stable signature extraction in larger cohorts compared with single-sample analyses. Drews module additionally provides a platinum-response classifier based on CIN signature activities, enabling a direct link between CN signatures and potential therapeutic response just for research purposes (for more details see Additional Details for Methods section, available as supplementary data at *Bioinformatics* online).

### 2.3 Benchmarking

To benchmark the performance and interpretability of the different signature frameworks implemented in PULPO, we carried out a two-level evaluation: (i) a theoretical comparison of the underlying catalogues and (ii) an empirical comparison based on our OGM ALL cohort.

At the theoretical level, we summarized the defining features and reported aetiologies of each CN signature from the COSMIC CN framework (Steele *et al.* 2022), the Tao catalogue (Tao *et al.* 2023), and the Drews CIN signatures (Drews *et al.* 2022) catalogue (Table 2, available as supplementary data at *Bioinformatics* online). For each signature, we collected the number of original feature space, the mechanism, the tool implemented for the analysis, the algorithm mainly used, the nomenclature and the number of signatures of each catalogue between others. These annotations were harmonized and compiled into a comparative reference table (Table 2, available as supplementary data at *Bioinformatics* online), which facilitated cross-referencing of signatures across catalogues and provided a theoretical baseline for interpreting the PULPO-derived signatures.

At the empirical level, we also summarized the success of each method in our cohort named success as the number of patients who get a mutational signature based on each catalogue (Table 3, available as supplementary data at *Bioinformatics* online). Additionally, a decomposition in each CN and SV signature catalogue is provided for four samples example table with the number of CNVs and SVs and its correspondence types (Table 4, available as supplementary data at *Bioinformatics* online).

### 2.4 Public dataset and external testing

To evaluate PULPO beyond OGM-based ALL, we analysed somatic CNVs and SVs from the open-access Open Pediatric Brain Tumor Atlas (OpenPBTA) cohort (Shapiro *et al.* 2023). CNVs and SVs calls were downloaded from the OpenPBTA release and reformatted into the CNV48 and SV32 feature spaces using dedicated preprocessing scripts that adapt these calls to the input format required by the PULPO CNV and SV processing modules (as for the OGM ALL cohort; scripts available on GitHub). All samples with CNV and/or SV calls were included in this external testing. In the case of CNVs, were tested using the three described above methodologies (Steele, Tao and Drews). Mutational signature extraction and exposure estimation were performed as described above for the OGM data. Unlike the OGM ALL cohort, OpenPBTA provides minor-allele copy-number information, which allowed us to classify segments with total copy number 2 into copy-number-neutral and loss-of-heterozygosity (LOH) events following the methodology stablish in Steele *et al.* (2022) only implemented in the COSMIC CN signatures framework. For the OGM data, where allele-specific copy-number information is not available, all segments with copy number 2 were treated as copy-number-neutral.

## 3 Results

To evaluate the feasibility and robustness of PULPO, we conducted a proof-of-concept analysis on 130 paediatric samples of patients diagnosed with acute lymphoblastic leukaemia (ALL). The samples were profiled using OGM technology (Bionano Genomics 2021) in conjunction with the Rare Variant Analysis pipeline. The use of ALL patient samples has been approved by the CEIm at Hospital Infantil Universitario Niño Jesús (HUNJ) under internal code R-0017/13 (approved on 8 May 2013), and written informed consent was obtained from all participants or their legal guardians, in accordance with the Declaration of Helsinki and applicable national regulations on biomedical research involving human subjects.

PULPO was executed on a standard computer equipped with 16 GB RAM, an 11th Gen Intel^®^ Core™ i7-1165G7 @ 2.80 GHz × 8 CPU, and Ubuntu 22.04.5 LTS, thereby demonstrating its compatibility with widely accessible computing infrastructures.

The pipeline successfully processed the majority of samples by automatically executing all the required steps for mutational signature extraction, generating standard outputs which include signature decomposition plots, reconstruction metrics, and comprehensive quality control summaries.

For most of the 130 samples analysed, the pipeline generated valid mutational signature profiles. In a minority of cases, however, mutational signatures could not be extracted because no CNVs (7/130) or SVs (3/130) were detected for those patients using OGM. These patients were excluded from the proof-of-concept analysis, resulting in a final cohort of 121 patients to facilitate downstream analyses.

The mean cosine similarity between observed and reconstructed COSMIC structural profiles across samples was 0.85 (range: 0.81–0.89), indicating accurate signature attribution (Tables 5 and 6, available as supplementary data at *Bioinformatics* online). Cosine similarity values below 0.8 are considered insufficient for confident assignments to know COSMIC structural signatures. Overall, these values support a high fidelity of signature decomposition.

For the 121-sample cohort, COSMIC signature SV7 was present by 100% of samples in the cohort (Fig. 1a, b, available as supplementary data at *Bioinformatics* online, Tables 5 and 7, available as supplementary data at *Bioinformatics* online). In contrast, the most frequent COSMIC CN signatures were CN9 (64.9% of samples) followed by CN25 (21.1% of samples) and CN20 (14% of samples) in terms of mutations assigned to each signature detected in the cohort (Fig. 1b and c, available as supplementary data at *Bioinformatics* online). Together, these results demonstrate that PULPO effectively automates COSMIC signature assignment on OGM data and captures mutational profiles consistent with known SV aetiologies even with different number of variants per sample (Fig. 2 and Table 8, available as supplementary data at *Bioinformatics* online).

Currently, there is no established reference pipeline for the extraction of COSMIC-like signatures from OGM-derived data, and to the best of our knowledge, PULPO is the first tool specifically designed to bridge this gap. Although the COSMIC mutational signature catalogue was not originally developed using OGM data, we posit that the high resolution and structural accuracy of OGM render it a compelling source for signature inference, especially for detecting large-scale genomic rearrangements that are often underrepresented in short-read sequencing data.

We next benchmarked PULPO across these three complementary CN signature frameworks (COSMIC CN, Drews and Tao/Sigminer) on the OGM ALL cohort (Table 9, available as supplementary data at *Bioinformatics* online). CN signature extraction successfully completed for all samples with both the COSMIC/Steele and Tao approaches (121/121, 100% success), whereas the Drews method assigned signatures to only 44/121 cases (36.36%), reflecting its more stringent requirements on input segmentation (Table 3, available as supplementary data at *Bioinformatics* online). COSMIC CN signatures were detected in 121 (100%) samples, with a mean of 1.20 signatures per sample, compared with 7.16 Drews signatures and 3 Tao signatures per sample, respectively. This benchmarking was restricted to CN catalogues, as the COSMIC SV signature catalogue currently represents the only widely used framework for SV signatures and, to the best of our knowledge, no alternative SV signature catalogues are available for systematic comparison. Taken together, these results indicate that PULPO can robustly accommodate multiple CN signature catalogues due to its modular design, while highlighting practical differences in sensitivity and applicability between methods when applied to real OGM data.

### 3.1 Testing OpenPBTA

The OpenPBTA CNV and SV profiles (*N* = 940) were successfully processed with PULPO, and CN signature extraction completed for all three frameworks (COSMIC CN, Drews and Tao). In total, 940 OpenPBTA samples were analysed, of which 940 (100%) exhibited at least one COSMIC CN signature with a range of 1–6 signatures per sample and a mean of 4 signatures, 940 (100%) at least one Drews signature with a range of 1–5 signatures and a mean of 3.5 signatures per sample and 940 (100%) at least one Tao signature with a range of 5–13 Tao signatures and a mean of 9.8 Tao signatures per sample. For SVs, 940 (100%) samples exhibited at least one COSMIC SV signature with a range of 1–5 signatures and a mean of 1.6 signatures per sample. In summary, for this cohort, the success rate is 100% for each method.

## 4 Discussion and conclusion

In this study, we demonstrate that PULPO enables a robust and accessible framework for SV signature analysis by leveraging high-resolution SV/CNV data generated via OGM and also NGS data. By systematically integrating these profiles with the COSMIC repository of mutational signatures (Alexandrov *et al.* 2020) through NMF-based decomposition and cosine similarity metrics, PULPO facilitates the accurate identification of structural mutational patterns across samples. By additionally integrating the Tao/Sigminer and Drews/CINSignatureQuantification CNV frameworks (Wang *et al.* 2021, Drews *et al.* 2022, Tao *et al.* 2023), PULPO provides a unified environment to compare multiple signature catalogues on the same data. Its implementation within a preconfigured Snakemake pipeline not only ensures reproducibility and scalability, but also significantly lowers the barrier to entry for researchers without extensive bioinformatics training. This integrative approach highlights the potential of OGM-based signature analysis as a complementary tool for genomic characterization, particularly in settings where conventional sequencing may overlook complex structural rearrangements.

PULPO presents significant potential for clinical translation by enabling the interpretation of structural mutational signatures in the context of underlying aetiological mechanisms. Through this approach, it contributes to a deeper understanding of the molecular processes driving tumorigenesis and supports the development of precision oncology strategies. Notably, the ability to associate specific signature profiles with therapeutic contexts—such as APOBEC activity or homologous recombination deficiency (HRD)—may inform drug repurposing efforts and highlight novel therapeutic vulnerabilities (Alexandrov *et al.* 2020, Watkins *et al.* 2023).

## 5 Limitations and future perspectives

Our benchmarking analyses indicate that most practical limitations arise from the underlying technologies and signature catalogues rather than from PULPO itself, although these factors likely exert a substantial influence on signature assignment. At the CNV level, COSMIC and Tao signatures could be assigned to all OGM samples, whereas Drews signatures were recovered only in a subset of cases (36.36%; Table 3, available as supplementary data at *Bioinformatics* online) (Drews *et al.* 2022, Steele *et al.* 2022, Tao *et al.* 2023). This behaviour appears intrinsic to the Drews catalogue, which was specifically designed to capture CIN-related copy-number processes, we view this as a strength, as the presence of Drews signatures in a given sample provides an additional, orthogonal check for CIN-like patterns on top of the COSMIC and Tao decompositions (Drews *et al.* 2022) (Table 4, available as supplementary data at *Bioinformatics* online). More generally, all three CNV catalogues were originally derived from non-OGM cohorts and may not fully capture paediatric or OGM-specific copy-number processes (Steele *et al.* 2022, Tao *et al.* 2023).

Several additional limitations are intrinsic to OGM. Despite the use of stringent filters, OGM can still produce false positives and false negatives and has known challenges in highly repetitive regions such as centromeres and telomeres, where structural events may be under-detected (Neveling *et al.* 2021). Intra-chromosomal rearrangements are annotated by Bionano Access as inversions or intra-chromosomal translocations (depending on the version), and PULPO deliberately preserves these labels rather than reclassifying them based on downstream assumptions. If Bionano Access mislabels a subset of intra-chromosomal events, this misclassification will propagate to the SV class counts used for signature assignment. Since COSMIC SV signatures distinguish inversions from translocations (Alexandrov *et al.* 2020), such errors could in principle influence the contribution of individual SV classes; however, intra-chromosomal translocations are relatively infrequent in our cohort, and we expect the impact on aggregate signature exposures to be modest. Improvements in OGM chemistry and algorithms are likely to alleviate some of these issues, and PULPO is designed to benefit from such updates without huge modifications to the workflow.

Together, these applications underscore PULPO’s potential to serve as a bridge between SV research and actionable clinical insights.

Looking forward, as OGM becomes more widely adopted and the volume of available datasets increases, we anticipate that *de novo* mutational signatures will be reconstructed directly from OGM-based cohorts. PULPO was developed with this future evolution in mind; its modular structure enables integration of updated COSMIC releases or alternative, OGM-specific signature catalogues with minimal adjustments, ensuring long-term utility and adaptability. Recent work employing bioinformatic methods to identify mutational signatures further supports the evolution of such approaches (Islam and Alexandrov 2021). This innovative integration not only enhances our understanding of the complex mutagenic processes driving cancer but also holds significant promise for clinical applications, including improved diagnostic accuracy, refined therapeutic stratification, and personalized treatment approaches.

## Supplementary Material

btag118_Supplementary_Data

## Code availability

The pipeline is open source and freely available under the MIT License at https://github.com/OncologyHNJ/PULPO-v.1.0 and DOI in Zenodo: https://zenodo.org/records/17749097.
