# Supplementary material for: PULPO: pipeline of understanding large-scale patterns of oncogenomic signatures
Source: Bioinformatics. 2026 Mar 10;42(6):btag118. doi: 10.1093/bioinformatics/btag118 (PMC13224964; doi:10.1093/bioinformatics/btag118)
Supplement: btag118_Supplementary_Data [file btag118_supplementary_data.docx]

Supplementary information

***Figures***





**Fig. S1. Overview of the contribution of SV and CN signatures in the cohort. a**, Nested donut plot summarising the proportion of mutations attributed to SV and CN signatures. The inner ring shows the global distribution of mutations assigned to SV versus CN signatures, whereas the outer ring displays the relative contribution of each individual signature within these two groups. **b**, Pie chart showing the proportion of mutations assigned to the SV signature detected in the ALL cohort. **c**, Pie chart showing the proportion of mutations assigned to each CN signature detected in the ALL cohort.


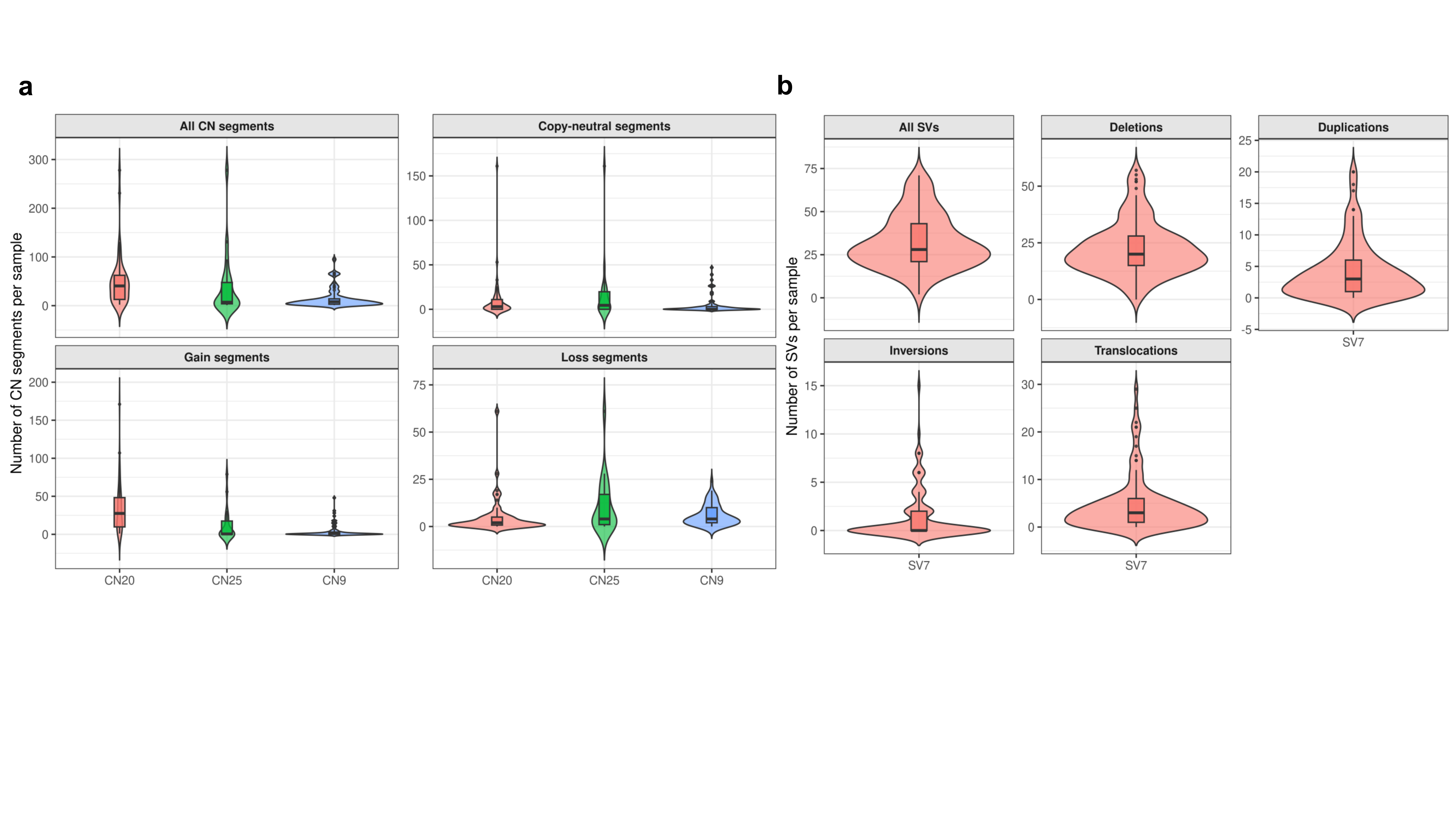
**Fig. S2. Violin-plot distributions of variants in the cohort in the signature-like-COSMIC. a,** Number of CNVs across the COSMIC CN signatures present in the cohort. **b,** Number of SVs across the COSMIC SV signatures present in the cohort.

***Tables***

**Table S1.** **SVs and CNVs filters applied to OGM data.**

| Category | Parameter | Value |
| --- | --- | --- |
| SVs filters | Feature SV overlap precision (Kbp) | 3 |
|  | SV masking filter | non masked |
|  | VAF filter | 0–1 |
|  | Self molecule count | 5 |
|  | % in control database | 0 |
|  | % in control database for enzyme | 0 |
|  | SV chimeric score | all |
|  | Found in self molecules | yes |
|  | Overlap genes | all |
| CNV filters | Feature CNV overlap precision (Kbp) | 15 |
|  | Copy number type | all |
|  | Copy number confidence | 0.99 |
|  | Copy number minimum size (bp) | 500,000 |
|  | Copy number masking filter | non masked |

**Footnote:**

Summary of the filters applied to CNVs and SVs OGM data.

**Table S2. Theoretical frame for each method**.

| Method | Features | Tool | Programming language | Mechanism | Algorithm | Pipeline | Number of signatures in the catalogue | Nomenclature | LOH |
| --- | --- | --- | --- | --- | --- | --- | --- | --- | --- |
| Steele | 176 | SigProfiler | Python and R package | Conceptual framework | NMF | Extraction + fitting | 48 | CN | Yes |
| Tao | 48 | Sigminer | R package | Agnostic | NMF | Extraction + fitting | 176 | CNS | Yes |
| Drews | 43 | CINSignatureQuantification | R package | Determinist | NNLS | Fitting | 43 | CX | No |

**Footnote:**

Comparative table of the theorical benchmark of each CN methodology included in PULPO. The highlights of each method are represented in each column.

**Table S3. OGM Cohort results for each method**.

| Method | Number of patients with signatures | Percentage of success in the cohort |
| --- | --- | --- |
| Steele/COSMIC | 121 | 100 % |
| Tao | 121 | 100 % |
| Drews | 44 | 36.36 % |

**Footnote:**

OGM Cohort results of each CN methodology present in PULPO. Percentage of success in the cohort meaning success the percentage of samples that get a mutational signature in the cohort.

**Table S4.** Four patients of the OGM cohort decomposition into different signatures catalogue and the number of variants of each one.

| Patient ID | SV COSMIC signatures | CN COSMIC signatures | Tao signatures | Drews signatures | CNVs segments | CNVs losses | CNVs gain | CNVs neutral | SVs | Del | Dup | Inv | Tra |
| --- | --- | --- | --- | --- | --- | --- | --- | --- | --- | --- | --- | --- | --- |
| Patient-69 | SV7 | CN20 | Sig1, Sig2, Sig3 | CX5, CX15, CX7, CX1 | 231 | 7 | 171 | 53 | 28 | 24 | 2 | 0 | 2 |
| Patient–60 | SV7 | CN9 | Sig1, Sig2 | CX3, CX1, CX5, CX7, CX10, CX2, CX14 | 65 | 16 | 10 | 39 | 29 | 20 | 5 | 0 | 4 |
| Patient-63 | SV7 | CN20, CN25 | Sig1, Sig2 | CX5, CX7, CX2, CX10, CX9, CX11, CX1 | 278 | 61 | 56 | 161 | 28 | 18 | 3 | 6 | 1 |
| Patient-34 | SV7 | CN9 | Sig1, Sig2, Sig3 | NA | 14 | 10 | 4 | 0 | 71 | 39 | 14 | 15 | 3 |

**Footnote:**

Data from four patients on cross-sectional variables of interest, including the number of structural variants and signatures extracted from each method.

**Table S5. Proportion of SV signatures in the cohort.**

| Signature | Percent |
| --- | --- |
| SV7 | 100 % (121/121) |

**Footnote:**

Summary of the SV signatures identified in the cohort (n = 121). Percentages indicate the proportion of samples in which each signature is present. SV7 was the only SV signature detected, being present in all samples.

**Table S6. Proportions of CN signatures and their combinations in the cohort.**

| Signature | Percent |
| --- | --- |
| CN9  CN20  CN25  CN9 + CN20  CN20 + CN25  CN9 + CN25  CN9 + CN20 + CN25 | 57 % (69/121)  46.3 % (56/121)  16.5 % (20/121)  11.57 % (14/121)  4.13 % (5/121)  2.48 % (3/121)  0.83 % (1/121) |

**Footnote:**

Summary of CN signatures and their co-occurrence patterns across the cohort (n = 121). Percentages indicate the proportion of samples in which each individual CN signature or signature combination is present. Each sample is counted once according to its specific combination of CN signatures.

**Table S7. Summary of genomic features for COSMIC SV signature detected in the cohort.**

| Signature | Type | Number of samples | Mean SVs per sample | Mean deletions | Mean duplications | Mean inversions | Mean translocations |
| --- | --- | --- | --- | --- | --- | --- | --- |
| SV7 | SV | 121 | 32.5 | 22.5 | 4.14 | 1.36 | 4.51 |

**Footnote:**

Summary of genomic characteristics of the COSMIC SV signature in the cohort including the mean of the cohort for the different kind of structural variants.

**Table S8. Summary of selected COSMIC SV and CN signatures detected in the cohort.**

| Signature | Catalogue | Proposed Aetiology | Potential therapeutic association | Number of samples |
| --- | --- | --- | --- | --- |
| SV7  CN9  CN20  CN25 | COSMIC SV32  COSMIC CN48  COSMIC CN48  COSMIC CN48 | Unknown  Chromosomal instability  Unknown  MSH6 inactivation and dMMR | Unknown  Unknown  Unknown  PD-1 blockade ***** | 121  69  56  20 |

**Footnote:**

Only COSMIC signatures detected in our cohort are shown. Proposed aetiologies and potential therapeutic associations were summarised from the original COSMIC catalogue (Degasperi et al., 2020; Steele et al., 2022). “*Unknown*” indicates that no specific mutational process or therapy has been conclusively established to date. *PD-1 blockade refers to immune checkpoint inhibition (e.g. pembrolizumab) clinically indicated in tumours with confirmed mismatch-repair deficiency/microsatellite instability (dMMR/MSI-high); CN25 itself is not currently a validated standalone predictive biomarker and should be interpreted in conjunction with standard MSI/dMMR testing* (Le et al., 2015)*.*

**Table S9. Summary of genomic features and overlap with alternative CN catalogues for COSMIC CN signatures detected in the cohort.**

| Signature | Type | Number of samples | Mean CN segments | Mean CN loss | Mean CN gain | Tao signatures | Drews signatures |
| --- | --- | --- | --- | --- | --- | --- | --- |
| CN9  CN20  CN25 | CN  CN  CN | 69  56  20 | 14.7  48.7  40.0 | 5.86  4.88  11.0 | 4.22  34.5  11.6 | 69  56  20 | 13  38  8 |

**Footnote:**

Summary of genomic characteristics of the COSMIC CN signatures in the cohort including the mean of the cohort for the different kind of copy number variants and the presence of others CN catalogues.

**Table S10.** **Tao method results.**

| De Novo Signature | Tao catalogue signature | Cosine similarity |
| --- | --- | --- |
| Sig1 | PCAWG_CNS12 | 0.891 |
| Sig2 | PCAWG_CNS8 | 0.614 |
| Sig3 | PACAWG_CNS11 | 0.816 |

**Footnote:** To unify comparations between methods, only that signatures with cosine similarity > 0.8 will be considered as matching signatures compared to the PCAWG Tao catalogue.

**Glossary of Key Terms**

*SV (structural variant).*

A genomic rearrangement involving DNA segments larger than a few dozen base pairs, including deletions, duplications, insertions, inversions, and translocations.

*SV signature.*

A characteristic pattern of structural variants defined by their type, size, and genomic context. In this study, SV signatures refer to the COSMIC SV signature catalogue unless otherwise specified.

*CNV (copy number variant).*

A genomic region showing a gain or loss in copy number relative to the normal diploid state, typically defined as a contiguous segment along the genome.

*CNA (copy number alteration).*

A somatic change in copy number in cancer cells, encompassing both focal and broad gains and losses; the term is commonly used in oncology-specific contexts.

*CN signature.*

A characteristic pattern of copy-number changes, defined across copy-number states, segment sizes, and breakpoint features. In this study, CN signatures refer to the COSMIC copy-number signature catalogue or alternative CNV signature frameworks where explicitly indicated.

**Additional details for Methods section:**

**Tao**

For the Tao framework (Tao *et al.*, 2023), CNV signatures were extracted using the implementation provided in the sigminer package (Wang et al., 2021). CNV segments were mapped to the X copy-number feature space, which jointly encodes absolute copy-number state, segment length, and the magnitude and symmetry of copy-number changes at segment breakpoints. Lacking the information of LOH events due to the lack of minor-allele information in OGM, all segments were analysed without adding LOH data.

Sample-wise counts for each 176 features were aggregated into a sample-by-feature matrix, after removing features and samples with zero total counts and replacing residual missing values with zero. De novo Tao CNV signatures were then inferred by non-negative matrix factorisation using the Brunet algorithm with multiple independent runs. The optimal number of signatures was selected based on the rank survey reported by sigminer (Wang et al., 2021), favouring solutions with high cophenetic correlation (>0.95) and high consensus silhouette scores (>0.7); when several ranks satisfied these quality criteria, the highest rank within this set was retained.

To annotate the extracted Tao signatures, we compared them against the CNS-specific reference copy-number catalogues distributed with sigminer. For this, each de novo signature profile was L1-normalised so that the entries within each column summed to one, and cosine similarities were computed against CNS_PCAWG176 reference catalogues. For every PULPO-derived Tao signature, we recorded the best-matching reference signature and its corresponding cosine similarity and summarised these matches in a similarity table (Supplementary Table S10), which facilitated the interpretation of our de novo signatures in the context of previously reported CN signatures.

**Drews**

For the Drews framework (Drews et al., 2022), cohort-level CN signature extraction was performed using the CINSignatureQuantification package. CNV segments were first reformatted into the input structure required by the quantifyCNSignatures() function (using a dedicated preprocessing script in PULPO) and harmonised across samples by standardising chromosome labels (mapping chromosomes 23 and 24 to X and Y, respectively) and sorting segments by sample, chromosome and genomic coordinate. To ensure sufficient chromosomal instability signal for robust signature estimation, we restricted the analysis to samples harbouring at least 5 CNVs, in line with recommendations for CIN-based signature quantification.

The cleaned cohort-level CNV table was then analysed with quantifyCNSignatures() (method = "drews", build = "hg38"), yielding sample-level activities for the Drews copy-number signatures together with associated feature matrices and experiment metadata. In addition, we applied the clinPredictionPlatinum() function to the resulting object to obtain the so-called platinum classifier, which provides probabilistic predictions of platinum sensitivity or resistance based on the inferred CIN signature activities. PULPO normalises and exports these predictions as a sample-wise table including the predicted class and, when available, per-class probabilities, and generates standard visualisations such as sample-by-component plots and activities heatmaps to facilitate interpretation. Finally, we recorded basic quality-control metrics (e.g. number of NA features per sample) and stored them as supplementary outputs, allowing users to assess data completeness and the robustness of the Drews-based CN signature analysis.
